# Physical and chemical mechanisms involved in adhesion of orthodontic bonding composites: in vitro evaluations

**DOI:** 10.1186/s12903-021-01715-9

**Published:** 2021-07-16

**Authors:** R. Condò, G. Mampieri, A. Cioffi, M. E. Cataldi, I. Frustaci, A. Giancotti, V. Campanella, V. Mussi, A. Convertino, L. Maiolo, G. Pasquantonio

**Affiliations:** 1 Department of Clinical Sciences and Translational Medicine, University of Rome “Tor Vergata”, Via Montpellier, 1, 00133 Rome, Italy; 2 PhD in Materials for Health, Environment and Energy, University of Rome “Tor Vergata”, Via della Ricerca Scientifica, 1, 00133 Rome, Italy; 3 Institute for Microelectronics and Microsystems - National Research Council, Unit of Rome, Via del Fosso del Cavaliere, 100, 00133 Rome, Italy

**Keywords:** Light-cure orthodontic composites, Shear bond strength, Field emission scanning electron microscope, Weight loss analysis and Raman spectroscope

## Abstract

**Background:**

Bond strength of orthodontic composite is strongly influenced by molecular and structural mechanisms. Aim of this in vitro study was to compare bond strength of light-cure orthodontic composites by measuring debonding forces and evaluating locations of bond failure. Investigations on chemical compositions clarified adhesive behaviors and abilities, exploring effects of ageing processes in this junction materials.

**Methods:**

Twelve enamel discs, from human premolars, were randomly coupled to one orthodontic adhesive system (Transbond XT™ 3 M UNITEK, USA, Light-Cure Orthodontic Paste, LEONE, Italy and Bisco Ortho Bracket Paste LC, BISCO, Illinois) and underwent to Shear Bond Strength test. Metallic brackets were bonded to twenty-seven human premolar, with one of the adhesive systems, to quantify, at FE-SEM magnifications, after debonding, the residual material on enamel and bracket base surfaces. Raman Spectroscopy analysis was performed on eight discs of each composites to investigate on chemical compositions, before and after accelerated aging procedures in human saliva and sugary drink.

**Results:**

Orthodontic adhesive systems showed similar strength of adhesion to enamel. The breakage of adhesive-adherent bond occurs in TXT at enamel-adhesive interface while in Bisco and Leone at adhesive-bracket interface. Accelerated in vitro aging demonstrated good physical–chemical stability for all composites, Bisco only, was weakly contaminated with respect to the other materials.

**Conclusion:**

A similar, clinically adequate and acceptable bond strength to enamel for debonding maneuvers was recorded in all orthodontic adhesive systems under examination. No significant chemical alterations are recorded, even in highly critical situations, not altering the initial mechanical properties of materials.

## Background

Adhesion can be defined as the sum of the chemical and physical forces that represent the molecular attraction between materials in close contact. It expresses the resistance to separation forces. Adhesion phenomena are critical in many clinical applications of dental materials, including orthodontic bonding. The success of the adhesion is strictly linked to the characteristics of the interfacing surfaces and to the properties of the material used as bonding. In orthodontics, polymeric adhesive resins are widely used as a dental bonding system to ensure an intimate and strong joint between the base of the bracket and the enamel surface [1].

An ideal adhesive system should have an optimal bond strength, i.e. able to withstand both chewing and orthodontic forces. At the same time, it should also allow an extremely easy and safe manual detachment of the bracket. This avoids the onset of permanent damage to the dental enamel and/or the persistence of residual material in situ, which is commonly removed mechanically with some manoeuvres, as drilling or air abrasion, able to produce alterations in the roughness of the tooth surface [2].

Currently, there is not uniformity of opinion on what is the optimal value of bond strength between direct bracket and tooth enamel [3–10]. Furthermore, a standard for assessing the bond strength to tooth enamel, or to other surfaces, of orthodontic adhesive systems is not clearly reported in the literature however, it is established that some factors must be considered, such as: the method of load application, the penetration speed, or crosshead speed, of the machinery used for the test (usually equal to 0.5 mm/min); the design of the bracket and the statistical analysis of the data [8]. I.R. Reynolds suggested a minimum value, i.e. valid for most of the clinical orthodontic needs, of the bond strength that orthodontic adhesives must have, it was estimated to be 6–8 MPa [3, 4]. Brantley and Eliades observed the existence of orthodontic adhesive systems whose shear bond strength can vary within an even greater range, that is, even between 8 and 30 MPa. Since that the most commonly used brackets have a hypothetical adhesion area of 16 mm^2^ and then, by measuring the force necessary to obtain the bracket debonding, which turned out to be on average 120 N, they were able to calculate the minimum value of the adhesion strength, i.e. equal to 7.5 MPa [8]. At the end of the treatment, the bond strength must be of such an extent as not to cause cracking or chipping (defects) on the surface of the dental enamel and prosthetic crowns [9]. For this reason, A.M. Compton reported that 7 MPa is the maximum value of adhesion to the enamel which would allow to avoid precisely the onset of such problems [10].

The best way to test a biomaterial is certainly its long-term clinical use, but also the in vitro measurement of detachment forces and adhesion plays an important role in the characterization of the adhesive potential of the orthodontic adhesive systems. In light-cured composites, the light-curing process does not always occur homogeneously throughout the material. The formation of radicals begins first on the surface exposed to sufficient light intensity. It is its fluidity that allows radicals to completely penetrate the entire structure of the material in order to carry out a complete radical cross-linking reaction. The bond strength of orthodontic adhesive composite used for bracket bonding appears to be strongly influenced by both molecular and structural mechanisms [11, 12].

Therefore, the aim of this in vitro study is to compare the bond strength of three different light-cure orthodontic adhesive resins by measuring the force of debonding and evaluating the location of bond failure. Subsequently, the chemical composition of the dispersed phase is investigated to explain how they affect the adhesive behaviour not only at the adhesive/enamel interface but at the adhesive/bracket base interface and how they are responsible for the ability of adhesion. To fully comprehend the material behaviour during its clinical use, we evaluate also the specimen properties after aging analysis in human saliva and sugary drink to understand if these effects can play a role in the alteration of the chemical structure of materials thus contributing in changing the adhesion properties of the resins.

## Methods

### Shear bond strength (SBS) test

Twelve human premolars, previously extracted, with the approval of the Ethics in Research Committee of the Centre of Health Sciences of the University of Rome “Tor Vergata”, for periodontal reasons, Italy, were selected, cleaned and stored in normal saline (0.9% NaCl) at 37 °C. For each tooth, the crown was separated from the respective roots below the amelo-cement junction, subsequently the cusps were removed with a 0.3 mm thick diamond separator disc, thus obtaining a section of enamel only, as thick as possible, with two surfaces perfectly parallel to each other. Each of the enamel sections obtained was inserted in the middle of a hollow plastic cylinder, with its occlusal face facing upwards. At this point, self-curing acrylic resin was poured into the cylinder until it was completely filled and taking great care not to cover the enamel section inserted. Once the acrylic resin had hardened, the sample was sawn and flattened to be flush with the tooth surface. The enamel disc was then finished first with a rubber cup and then with a silicon carbide polishing paper used, after softening with water.

Each sample was placed in a mold (10 pk in volume) to allow the correct compaction of the orthodontic resins. Each disc obtained was inserted horizontally inside a support with screws, which had a surmounted cylinder on its upper part that allowed the adhesive orthodontic resin to be perfectly in contact with the surface of the enamel, so as to create, precisely adhered to it, an orthodontic resin cylinder of a suitable diameter for testing with Ultra Tester Machine 91099/KB3 (Ultradent).

The twelve disks obtained were randomly divided in three groups (n = 12) and each group was randomly assigned a different adhesive system used for bracket bonding (Table 1).

**Table 1:** Light-cured orthodontic adhesive systems considered in the study

	Orthodontic adhesive system	Manufacturer
TXT	Transbond XT™ Light Cure Adhesive is composed of a primer in bottle and a paste in syringe, 37% orthophosphoric acid (ETCH-37TM, BISCO) in add	3 M Unitek, Monrovia, CA, USA
LEONE	Light-Cure Orthodontic Paste is composed of a paste in syringe (F3172-01), a primer in bottle (F3171-01) and an acid etch (F3143-01)	Leone spa, Sesto Fiorentino. FI, Italy
BISCO	Bisco Ortho Bracket Paste LC is composed of a paste in syringe, a one-step primer in bottle and an etching gel (ETCH-37TM, BISCO) in syringe	Bisco, Schaumburg, Illinois, USA

The enamel disc was first cleaned with silicon carbide polishing paper and water and then dried with a jet of air. Etching gel was applied to the tooth surface for 30 s. After rinsing and air-drying, a uniform layer of primer was applied with a brush on the surface to be bonded, using circular movements, for 30 s. The primer was polymerized with the lamp for 30 s. The disk was inserted in the special support and the adhesive resin was brought into the cylindrical mould by a small spatula and compacted with a ball shutter. Finally, polymerization was carried out with a VALO curing light (Ultradent), according to the times indicated by the manufacturer, from top and side.

The samples, after 12 h stored in normal saline (0.9% NaCl) at 37 °C, were inserted, one at a time, vertically inside a metal support. The machine was equipped by a holder to locate the samples and a stab that applies a shear stress force over the cylindrical sample until reaching bind rupture, obtaining the maximum tensile shear force.

Each enamel disc was involved in the test up to 3 times. Each measurement was performed after removing the residual adhesive material from the sample and also the thin layer of enamel interfaced with it and, only after reconstituting the sample, according to the preparation protocol. Twelve measurements for each orthodontic adhesive system were obtained stressing under tension until failure using a crosshead speed of 1 mm/min. At the end of the test it is possible to see on the display of the machine the peak of force to which the material has resisted before detaching from the enamel surface.

### Field emission scanning electron microscopy (FE-SEM) characterization

Twenty-seven human premolars, extracted with the approval of the Ethics in Research Committee of the Centre of Health Sciences of the University of Rome “Tor Vergata”, for periodontal reasons, were selected, cleaned, stored in normal saline (0.9% NaCl) at 37 °C and subsequently randomly divided into three groups (n = 9), each of which was coupled at one of the three orthodontic adhesive systems under examination. The root of each premolar was inserted, until reaching the amelo-cement junction, inside a base prepared with Tenatex red wax (Kemdent), to better ensure the stability of the tooth during bonding manoeuvres. The operative procedure involved the initial cleaning of the enamel surface with pumice and water, followed by drying with a jet of air.

Then, Ovation (Densply GAC International, Bohemia, NY, USA) stainless steel brackets equipped with 3-layer Supermesh base were tested. Each bracket was bonded, always by same operator, to the vestibular surface of the assigned tooth, respecting the orthodontic direct bonding protocol provided by the adhesive material manufacturer.

All the orthodontic adhesive systems have been light-activated as recommended by each producer, applying a light-curing unit (LCU) (LED Starlight lamp), whose power density was previously measured with a curing radiometer (Model 100, Demetron Research Corp. Serial No. 129540) and then set at 400 mW/cm^2^, according to the pulse-delay light-curing methodology and placing the tip of the light unit at a minimum distance of 1.0 mm from the dental enamel surface.

All the specimens obtained were stored in normal saline (0.9% NaCl) at 37 °C for 24 h, in order to facilitate the polymerization and the hydration of both the material and the tooth. The same operator also carried out the detachment of the brackets from the surfaces of the tooth enamel, carefully inserting the working part of a stainless steel ligature pliers (P 1919-00, Leone spa), in the interface between the metal base of the bracket and the orthodontic adhesive resin, making sure to reproduce always the same twisting motion. Morphological investigations of the brackets and premolars on which they were previously attached, were carried out through image acquisitions, from 50 to 60×, obtained by Leo Supra 35 FE-SEM Field Emission Scanning Electron Microscope (Carl Zeiss, Germany).

### Weight loss analysis

With a thermo-formed polyurethane mould, previously made that reproduced inside 3 negative discs (10.0 mm in diameter and 4.0 mm thick), specimens of each material to be tested were obtained. Each disc has been completely filled with one of the three orthodontic resins considered in the study and before the polymerization process, a transparent strip (Hawe Neos Dental, Bioggio, Switzerland) has been placed and pressed to create the smoother and more uniform surface as possible. Glass plates were placed on the top and bottom of the mould to provide flat surfaces.

A light-curing unit (LCU) (LED Starlight lamp) has been used, whose power density was previously measured with a curing radiometer (Model 100, Demetron Research Corp. Serial No. 129540) and then set at 400 mW/cm^2^. The orthodontic composite resins have been light-activated as recommended by each manufacturer, applying the LCU at the top and bottom surfaces, where the light tip was placed in contact with the glass plate at a distance of 1.0 mm from the specimens.

Eight test discs (10.0 mm in diameter and 4.0 mm in thick), made of each orthodontic resin in exam, have been obtained for a total of 24 specimens. Samples were divided into 2 randomly groups (n = 12) and stored in distilled water at 37 °C until they have been used for the study of the accelerated aging effects to verify the chemical stability of the materials. For this purpose, one group of samples were immersed in human saliva and the other group in a sugary drink at predetermined times of 1, 7, 14, 21 and 28 days in two different solutions able to simulate the hostile environment of the oral cavity: sugary drink and human saliva (Table 2).

**Table 2:** Ageing solutions considered in the study

Testing solutions	Description
Sugary drink	5 ml of Coke ®
Saliva	5 ml human saliva collected according to standard protocols from healthy male volunteers, no smokers or drinkers, aged between 25 and 40 years

Each sample was placed into an Eppendorf test tube, immersed in its corresponding ageing solution at fixed temperature of 37 °C, at established time intervals, until one month of storage have been got. Different samples for each material have been tested and weighted 5 times with a precision balance (Mettler Toledo) after each time interval. For each weighing, the samples were pulled out from the tube, rinsed in deionized water for 5 min and dried in a nitrogen flux. Furthermore, before the measurement phase, they were stored 3 h in a glow box at 30 °C and 30% of humidity, to promote the complete water evaporation on the composite materials. During the handling of the samples, a Teflon tweezer was used which ensured to avoid any accidental damage or any change to the resins surfaces. In order to compare samples different in size and weight the percentage weight relative variation has been calculated by the relative weight shift: (Final weight—Initial weight)/Initial weight. Finally, the average and the standard deviation of the relative percentage weight variations have been got.

### Raman spectroscopy analysis

A Thermo Scientific DXR Raman Microscope has been used to investigate the chemical composition of the materials before and after the ageing procedure in saliva and sugary drink. A 532 nm laser source powered at 10 mW with an exposure time of t = 1 s for 200 accumulations was employed. The spectra were acquired in the range 300–3300 cm^−1^ with a 50 × objective and a 5th order polynomial correction was used to correct for fluorescence contribution.

### Statistical analysis

The data obtained by SBS test and Weight loss analisys, were analysed using Kruskal–Wallis and Mann–Whitney-U tests; Bonferroni Scheffe, and Sidak multiple comparison tests were used, p values were computed and compared with statistical significance at the p = 0.05 level. The data were analysed with the statistical software STATA (STATA Statistical Software release 12.1; Stata Corporation, College Station, TX). FE-SEM characterization data were analysed in a descriptive way, while the results of Raman Spectroscopy analysis were interpreted in a qualitative mode.

## Results

### SBS test results

The collected data have been summarized in Table 3, which indicates, for each orthodontic adhesive system under examination, the SBS values, expressed in newton (N).

**Table 3:** Shear bond strength test values on the three orthodontic adhesive system examined

Measurement	TXT SBS (N)	LEONE SBS (N)	BISCO SBS (N)
n. 1	20.4	7.4	4.2
n. 2	19.2	5.6	13.3
n.3	10.1	17.2	12.5
n. 4	18.9	9.9	16.1
n. 5	17.6	9.9	12.3
n. 6	9.5	9.4	14.9
n. 7	17	11	10.2
n. 8	6.5	6.1	8.6
n. 9	17.6	8.8	23.2
n. 10	13.9	15.3	14
n. 11	5.9	10.4	14
n. 12	11.3	7.3	10.6

Statistical analysis of the values obtained from the 12 measurements performed on each material has been executed and the average, standard deviation (DS), minimum (Min) and maximum (Max) values of SBS, expressed in megapascal (MPa), have been shown (Table 4). Differences between groups were evaluated using a t statistic at the p = 0.05.

**Table 4:** Average, standard deviation (DS), minimum (Min) and maximum (Max) values of SBS, expressed in MPa

Orthodontic adhesive system	Average SBS (MPa)	DS SBS (MPa)	Min SBS (MPa)	Max SBS (MPa)
TXT	**4.45**	1.32	1.88	6.49
Leone	**3.14**	1.51	1.78	5.47
Bisco	**4.08**	1.73	1.34	7.38

Bold values indicate to better highlight the SBS values that result from the average obtained between the minimum and maximum SBS values that have been recorded

Specifically, the values in N describe the SBS considering the surface of the retentive base, while the values in MPa, obtained by dividing the values in newton by the base areas, exclude the influence of the millimetre adhesion of the base and strictly reflect the effectiveness of the retention mechanism.

**Fig. 1:**
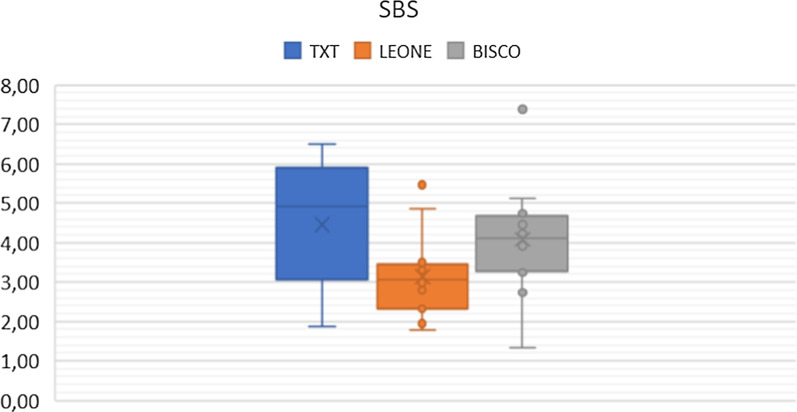
Scatter plot of the SBS values of the three orthodontic adhesive systems, expressed in MPa

The average SBS obtained is equal to 4.45 MPa for TXT, to 3.14 MPa for Leone and to 4.08 MPa for Bisco (Fig. 1).

### SEM characterization results

**Fig. 2:**
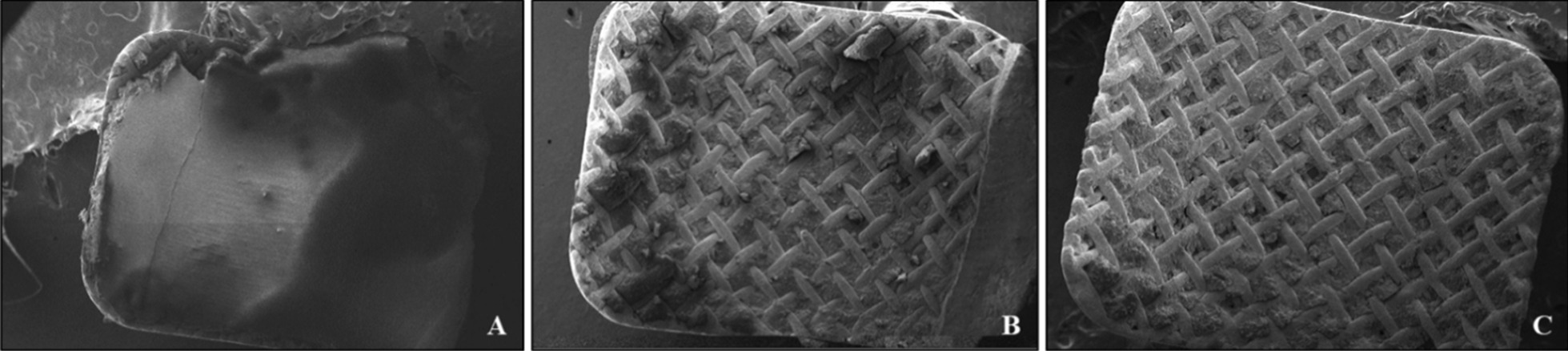
SEM magnifications relating to the material residues of the three orthodontic adhesive systems that remain on the base of the brackets after debonding, in particular it is observed in **a** TXT (51×), **b** Leone (54×) and finally **c** Bisco (59×)

The most significant images obtained at SEM were found to be those relating to the residual adhesive material on the base of the brackets and are illustrated in Fig. 2a–c.

Figure 2 illustrates the most frequently observed behaviour for each orthodontic adhesive system and significant differences between TXT and the other two resins can be noted. The images show that the breakage of the adhesive-adherent bond occurs, for the TXT, at the enamel-adhesive interface, given the large amount of resin residual on the base of the bracket (Fig. 2a). The situation in Bisco (Fig. 2c) appears different: given the scarce remaining adhesive material on the retina of the bracket base, is evident that the detachment at the adhesive-attachment interface occurred. Finally, the Leone (Fig. 2b), shows a more similar performance to Bisco adhesive resin.

In most of the samples examined, FE-SEM images show that the 3-layer Supermesh net of the Ovation bracket is able to retain a good amount of adhesive resin between its meshes.

### Weight loss analysis results

The different adhesive materials have been stored for one month in sugary drink or saliva and chemical stability and weight losses have been determined. After each time intervals, each sample has been weighted at least 5 times and we calculated the mean value. The statistical analysis has been performed, obtaining a p value of 0.05 for the significance of the used data. In any case, none of the specimens have shown significate weight loss values, thus proving a good material stability.

As expected, it was registered a slight increase in their weight for all the materials due to the absorption of organic residues in the vial during the samples storage.

**Fig.3:**
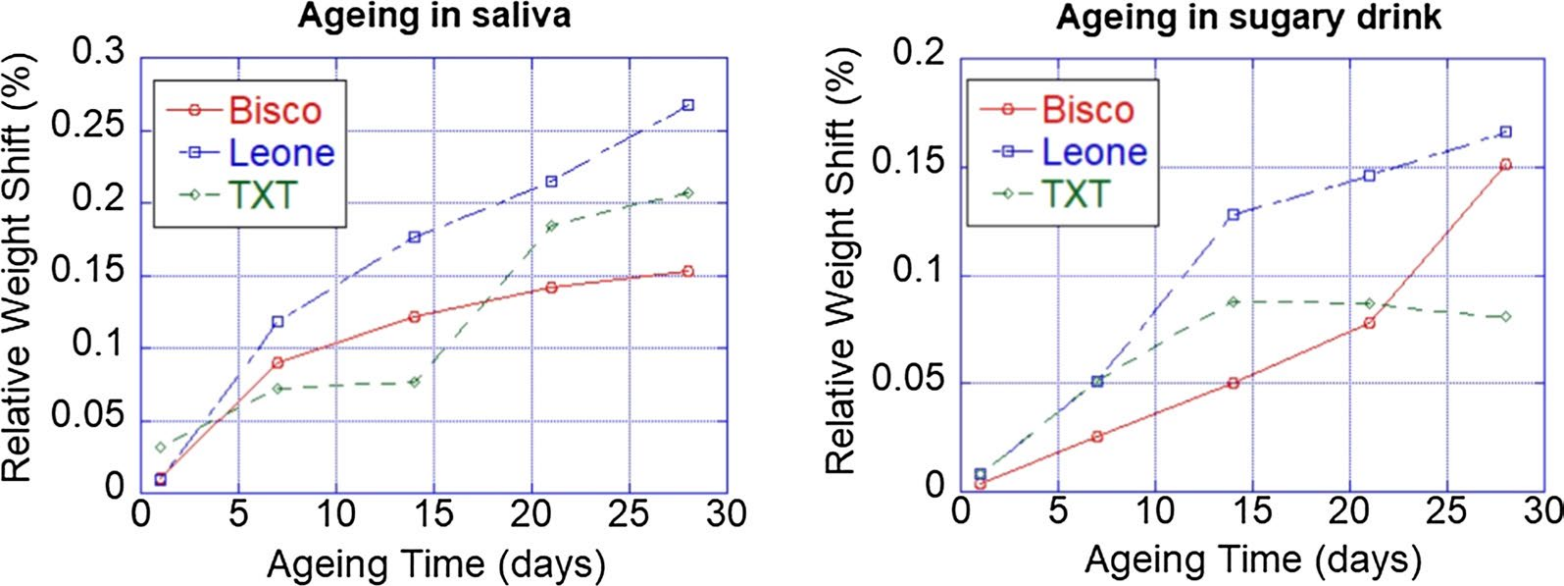
**a** The relative weight shifts for the three orthodontic adhesive systems samples stored in human saliva; **b** the relative weight shifts for the three orthodontic adhesive systems samples stored in sugary drink

An interesting argue is related to the saturation of this weight change depending by the type of storing liquid and by the time. In Fig. 3, it is possible to observe the relative weight shift for the samples stored in saliva and sugary drink for the different materials.

In saliva ageing (Fig. 3a), Bisco and Leone resins demonstrate a very analogous pattern remaining stable in the first period of in saliva immersion and then increasing, about 2 × 10^−4^ g/6 × 10^−4^ g in weight, at regular intervals of 5 and 15 days. They show a very similar trend with a monotone increasing while, in TXT is possible to observe a plateau up to two weeks and a following increasing of the weight. The interaction of TXT resin with human saliva showed a different model trend (Fig. 3a), by which, during the first 5 days of immersion, it tended to arise its weight of 4 × 10^−4^ g and then remains almost steady over the next 10 days. This second monotone phase then changes rapidly over the last 15 days, with a sudden increase in weight, in the order of 1 × 10^−3^ g. This behaviour can be related to a change in the absorption of TXT during the ageing. In any case, after one month Bisco exhibits the most stable performance.

For ageing in sugary drink (Fig. 3b), Bisco and Leone resins have a total weight increase of 1 × 10^−3^ g, which is regular in all the 30 days. Every 5–10 days, there was an increase of 2 × 10^−4^ g in both samples, with a starting tendency to a stabilization, as indicated by the Weight/Aging curve. The TXT resin, instead, interacted differently with the sugary drink: during the first 15 days of immersion, weight increase has been recorded at regular intervals of 2 × 10^−3^ g every 7 days. Subsequently, it has been characterized by an enhancement of 6 × 10^−3^ g over the last 15 days.

### Raman spectroscopy analysis results

**Fig. 4:**
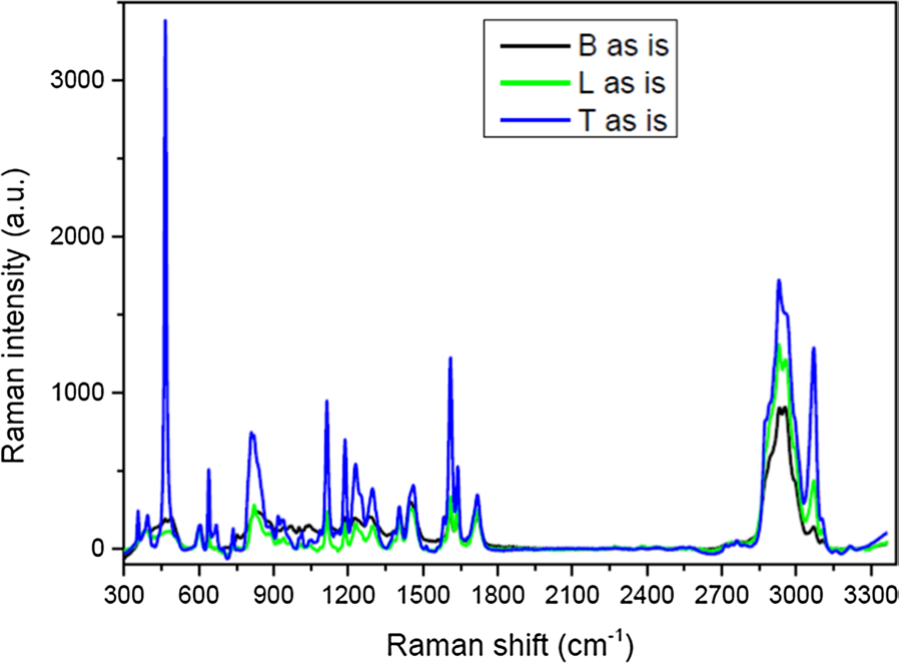
Comparison among the Raman spectra of the three investigated materials

In Fig. 4 the comparison of the Raman spectra of the composition of the three orthodontic adhesive resins has been illustrated.

**Fig. 5:**
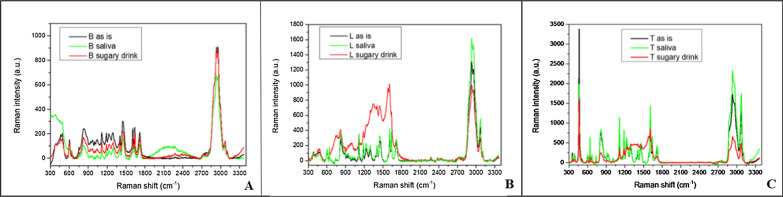
**a** Comparison between the Raman spectra collected on Bisco before and after ageing in saliva and sugary drink; **b** comparison between the Raman spectra collected on Leone before and after ageing in saliva and sugary drink; **c** comparison between the Raman spectra collected on Transbond XT before and after ageing in saliva and sugary drink

In Fig. 5 the comparison between the Raman spectra collected on Bisco (Fig. 5a), Leone (Fig. 5b) and TXT (Fig. 5c) before and after ageing in saliva and sugary drink has been presented.

In particular, for ageing in saliva, Leone and TXT do not show spectral variations, while Bisco presents a small intensity increase due to the presence of organic residues in the region between 1800 and 2700 cm^−1^. Conversely, in case of ageing in sugary drink, Bisco does not reveal any variation in the spectrum while Leone and TXT show new contributions and a broad spectral convolution in the region 1200–1800 cm^−1^ due to surface accumulation of organics (Fig. 5a, b).

## Discussion

The bond strength, that the orthodontic adhesive system is able to generate, influences the outcome of the bracket bonding to the surface of the dental enamel. This is essential for the success of the treatment since, from a clinical point of view, phenomena of accidental debonding could be the cause of damage to the dental enamel, increase the number of appointments necessary and/or extend both the operating times and those necessary to complete the orthodontic therapy [13]. Specifically, the incidence of the detachment phenomena was described both at the enamel—adhesive system interface and at the adhesive system—bracket base interface. It has been observed that these phenomena depend on the value of the bond strength of the orthodontic adhesive, but are influenced also by high mechanical stresses that occur during the orthodontic therapy, or as a result of a decrease in the bond strength at the interface, as occurs when using brackets in polymeric material [8].

F.L. Romano evaluated in vivo the failure rate of adhesion of metal brackets to the tooth enamel surface of both arches with the TXT adhesive system. It was found to be equal to 1.57%, i.e. only 3 brackets out of 190 in a 6-month period underwent accidental debonding [14]. These data are indicative of why this material is, to date, the most widely used adhesive system, as a control, both in clinical and laboratory studies and why TXT has been considered in our in vitro study, despite the abundant scientific literature already provided on it [15–22].

In order to investigate the orthodontic bond strength, the majority of researchers used the strength of the "shear" bond rather than tension or torsion, as the former was found to be the most reproducible. As regards the values ​​of the orthodontic bond strength, reported in the literature, it is actually observed that they can vary considerably also depending on the specimen preparation specifications and the tests conditions [23–34].

C. Sturdevant has shown that, depending on the material of which the bracket is made of, the value of the bond strength of the adhesive system, obtained following mechanical tests of resistance to shear strengths, can vary between 17 and 24 MPa [35]. In this regard, T.R. Katona has conducted extensive researches demonstrating that this simple approach of measuring the strength of adhesion, can provide often incorrect results [36]. Indeed, the stress produced on the bracket and on the enamel is not homogeneous, but it is concentrated, generating a local stress greater than that created between the applied force and the interface. The result is an underestimation of the local stress, that causes the failure of the adhesive bond, caused by micro-cracks propagation through the adhesive itself, which is more fragile and, probably, also through one or both interfaces [37, 38].

N. Fox hypothesized an existing relationship between the force application site and the surface of the base of the bracket, noting that a variability in the arrangement of this site and the relative position of the constituents of the adhesion system (enamel-adhesive-bracket), is able to determine substantial differences in the measurement of that force which is responsible for the failure of the adhesive bond [39].

Results of tests with tensile or shear strength can determine a coefficient of variation or relative standard deviation [(standard deviation/mean) × 100%] ranging from 20 to 30%. Typically, tensile force tests produce a lower coefficient of variation than the more common shear strength analysis [8].

By comparing the data obtained in our study, it is possible to highlight how all the adhesive systems under examination provide average SBS values in the range of 3—4.5 MPa. As shown in Table 4, TXT, with an average SBS value equal to 4.45 MPa, was found to be the adhesive material with the greatest adhesion strength, on the contrary Leone proved to have the lowest average SBS values, in fact equal to 3.14 MPa. The Bisco, on the other hand, showed intermediate SBS average values, equal to 4.08 MPa, but with larger variations from the lowest (1.34 MPa) to the highest (7.38 MPa) measurement among all the samples examined (Table 4).

However, it is important to underline that all the data collected by us are far from the parameters suggested by I.R. Reynolds as clinically acceptable levels of adhesion strength, i.e. 6–8 MPa [40]. Various studies have suggested bond strengths between 2.8 and 10 MPa to be clinically adequate [4, 5]. The same comparison, concerning the TXT and the SBS parameters present in the literature, shows an important difference in the results obtained in our study (Table 4) [41]. This significant discrepancy in the results is most likely explained by the fact that our study was purposely conducted without the use of brackets since, from the literature it is clear that the differing geometries of the bases of the bracket are able to greatly influence the strength of adhesion of orthodontic adhesive systems [41]. However, the purpose of our study was to compare the adhesion capacity of the three orthodontic adhesive systems in question and for this reason we chose to isolate the "bracket base" variable, focusing attention on the real resistance that the resin alone offers shear forces.

The objective of many studies in the literature is to demonstrate, following the detachment of the bracket, at what level the breaking of the bond of the bracket to the tooth surface occurs. When testing for adhesive bond failure, three situations may arise: bond failure at the base bracket/adhesive interface, at the adhesive/enamel interface, or cohesive failure [42]. The literature shows that the fracture gap can be localized, to the same extent, both at the enamel/adhesive interface, and at the bracket/adhesive interface [43–49].

The orthodontic adhesive resin however remaining on the enamel surface, after the bracket debonding, is necessarily mechanically removed from the tooth surface by milling and this entails the risk of accidentally removing even the most superficial layer of the dental tissue [25].

According to S. Elekdag-Turk, the prevalence of adhesive bond failure at the bracket/resin interface becomes a protective phenomenon for the enamel, precisely because, at the moment of detachment, this structure remains intact, preventing damage such as the loss of superficial tissue fragments therefore, cleaning the dental surface from the residual adhesive resin is perhaps less risky than the damage induced by bracket debonding [50]. Regarding the breaking of the bond at the enamel/adhesive interface, according to T. De Melo, the anatomy, the curvature and the design of the base of the bracket, may be the factors responsible for the greater strength of the bonding at bracket/adhesive interface and is just this condition to favour the major preservation of the dental enamel, because only a thin layer of residual material remains to be removed by milling [51].

It is also true that the removal of a bracket attached to the tooth through a high SBS adhesive system can increase the incidence of fractures or micro-cracks affecting the enamel, because of the increased effort have to be spend to remove the retained adhesive resin from the enamel surface [25]. Actually, what is considered as desirable in the debonding milling manoeuvres is to remove the bracket without damaging the enamel surface. It was also quantified that even the safest debonding manoeuvres were found to be responsible for the loss of about 10–20 µm of surface enamel [8].

The greater SBS to dental enamel demonstrated by TXT, could increase the probability of iatrogenic lesions of the hard tissues of the tooth occurring during the manoeuvre of bracket debonding. On the other hand, the greater affinity for chemical bonding, or cohesion, of TXT for the metal surface of the base of the bracket, would guarantee the orthodontist better holding performance of the bracket, during the active phases of the treatment.

The FE-SEM analysis (Fig. 2) of the bracket bases coupled at one of the three orthodontic adhesive systems under examination, has identified the adhesive interface in which the fracture and detachment of the bracket most commonly occurred. As regards TXT, the fracture occurs at the enamel-adhesive interface, given the large amount of resin residual on the base of the bracket (Fig. 2a). Highly filled resin composites have been observed to bond to mechanically retained metal brackets better than lightly filled composites [52]. Which once again TXT is the material repeatedly subjected to tests, some of the studies in the literature presented showed a tendency for the adhesive bond to break mainly at level of the enamel-adhesive interface, rather than the adhesive-bracket one [25, 27, 28, 30, 53]. This latter evidence is precisely in accordance with the results obtained in our in vitro study.

It has been reported that, enhancement of bond strength may compromise safe bonding, in fact, the detachment of a large part of TXT from the dental surface could expose, even if in a minimal percentage (1–30%), the enamel to damages such as micro-fractures or loss of superficial hard tissue. Bisco and Leone, which have instead shown a greater tendency to remain adherent to the enamel surface, have to be mechanically removed from the tooth by mechanical milling, taking care not to damage the dental enamel. It has been observed that, the resin tags that remain on the enamel surface, for a long time after the bracket debonding, can change colour as well as constitute sites for bacterial adherence [54]. Finally, the FE-SEM images however show a good performance of the Ovation bracket in most of the samples examined.

The investigation of aging in saliva and in sugary drink has been performed with the intent of establishing whether the recorded dimensional variations, even if minimal, were still capable of altering the physical–chemical properties of the surface of the three orthodontic composite resins under examination. Data on weight changes in saliva, Fig. 3a, showed a small weight increase in the order of 10^−4^ g, which is however considered not significant in terms of the chemical stability of the examined materials. These minimal modifications in weight values can be expected and caused by the surface adsorption phenomenon of organic saliva residues, proteins and mucus, as the result of reversible interactions, as it does not imply any type of irreversible chemical reaction as can be seen also by the unchanged colour of the sample surface. Overall, this enhancement in weighting resulted not uniform in the trends belonged to each orthodontic adhesive resins, although it showed a tendency to stabilize over time. The investigation of ageing in saliva, it has been followed that the minimal dimensional variation recorded in the tested orthodontic resins does not able to alter physical–chemical properties in the surface.

As showed in Fig. 3b, which summarizes the weight measurements of the samples over 30 days, even in this case the trends of Bisco and Leone resins can be overlapping, a similar trend for them is shown, while TXT reveals a weight saturation after fifteen days, thus demonstrating a different type on interaction with the testing solution. About weight changes for samples stored in sugary drink, it is showed a similar behaviour than which one recorded in saliva: a different interaction of the TXT is recorded both in saliva than in the sugary drink respect Bisco and Leone adhesive resins.

Overall, the weight changes of the three materials are again considered almost negligible and no materials losses can be evaluated in all the tests; therefore, all the orthodontic resins have shown to be resistant to the acidic and corrosive components of the sugary drink used in the experiment.

Raman Spectroscopy was a powerful tool, it has been used to investigate on the chemical composition of the three orthodontic adhesive resins, especially on the nature of their dispersed phase and, to establish the effects that occur following phenomena of aging in saliva and sugary drink, precisely at the level of the surface. Therefore, the purpose of this analysis has been twofold: a simple compositional comparison of the three adhesive resins (Fig. 3b) and an evaluation of the quality of the three materials (Fig. 4) in response to an exposure, until one month, in two different aging solutions to likely correlate these findings to possible changes in mechanical behaviour of the materials. On the basis of the chemical bonds reproduced in the Raman spectra it has been established that the type of filler prevalent in the dispersed phase of each resin is quartz. Strong affinities between the spectra of Bisco and Leone have been revealed, while the TXT exhibited a peculiar peak at 450 cm^−1^ associated to the higher percentage of quartz in its chemical composition (Fig. 4), as also reported by its safety data sheet. This last data allowed to justify both the diversity in the adhesive behaviour of the TXT resin and the similarities of performances demonstrated by the Bisco and Leone resins and finally, to justify the greater chemical affinity, or cohesion force which, following debonding, the TXT showed (Fig. 2a) with the metal interface represented by the base of the Ovation bracket, compared to the other two orthodontic resins tested (Fig. 2b, c). It is known that a higher content in fused quartz fillers, involves the attainment of high compressive strength and stiffness, the abrasion resistance and, the reduction the thermal dimensional change of the resin to a value matching that of tooth structure, effectively increasing adhesion to both the interfaces: adhesive/enamel and adhesive/base bracket. The surface profile and microstructure of the orthodontic composites are subjected to changes arising from degradation and wear processes in service; through Raman analysis it was observed that, the ageing in saliva usually produces the appearance of new broad bands in the region ranging from 900 to 2500 cm^−1^ ascribed to a possible fluorescence signal coming from the adsorption of organic residues [55]. Here, all the three materials demonstrate exhibit a good behaviour showing small changes in the spectra without the presence of pronounced new bands, thus indicating the absence of relevant and permanent surface effects (Fig. 5). Conversely, in case of ageing in sugary drink, only Bisco does not reveal any variation in its spectrum. Therefore, Raman analysis on samples stored in saliva and sugary drink highlighted that, Bisco is weakly contaminated with respect to the other two materials, and we speculate that this could be associated to peculiar moisture resistance properties.

## Conclusions

In all the orthodontic adhesive systems under examination, a rather similar bond strength to the dental enamel is recorded, clinically adequate and acceptable for debonding manoeuvres. Breakage of the adhesive-adherent bond occurs for TXT at the enamel-adhesive interface and conversely, for Bisco and Leone, it occurs mainly at the adhesive-bracket interface. All the resins are stable in human saliva, which is not able to alter the physicochemical properties of surface and they are also resistant to the acid and corrosive components of the sweetened drink used in the experiment. This testifies to the good biocompatibility of these resinous polymers, given their chemical stability even in highly critical situations. Raman analysis shows that no significant chemical alterations are observed on the resins, thus not altering the initial mechanical properties of these materials of and therefore also the expected clinical performances.

## Data Availability

The datasets used and/or analyzed during the current study are available from the corresponding author on reasonable request.

## Abbreviations

FE-SEM: Field emission scanning electron microscopes
TXT: Transbond XT™ light cure adhesive
Bisco: Light-cure orthodontic paste
Leone: Bisco ortho bracket paste LC
i.e.: Id est
MPa: Megapascal
N: Newton
kg: Kilogram
lb: Libra
mm: Millimeter
°C: Degree celsius
SBS: Shear bond strength
Tab.: Table
n: Number
MW: Megawatt
cm: Centimetre
LCU: Light-curing unit
ml: Milliliter
%: Percent
t: Time
s: Second
DS: Standard deviation
Min: Minimum
Max: Maximum
Fig.: Figure
a.u.: Arbitrary unit
min: Minute
µm: Micron
g: Gram
